# Complosome Regulates Hematopoiesis at the Mitochondria Level

**DOI:** 10.1007/s12015-025-10856-1

**Published:** 2025-03-07

**Authors:** Adrian Konopko, Agnieszka Łukomska, Janina Ratajczak, Magdalena Kucia, Mariusz Z. Ratajczak

**Affiliations:** 1 Center for Preclinical Studies and Technology, Department of Regenerative Medicine, Warsaw Medical University, Warsaw, Poland; 2 Stem Cell Institute at Graham Brown Cancer Center, University of Louisville, 500 S. Floyd Street, Rm. 107, Louisville, KY 40202 USA

**Keywords:** Complosome, Mitochondria, Hematopoiesis, Glycolysis, Glucose uptake

## Abstract

The intracellular complement network, known as the complosome, regulates lymphocyte biology, which is well established. Recently, however, we demonstrated that the complosome is also expressed in hematopoietic stem/progenitor cells (HSPCs) in addition to lymphocytes. In our previous work, murine lineage-negative (Lin^−^) bone marrow (BM) mononuclear cells (BMMNC) from mice lacking the intracellular C3 and C5 complosome proteins displayed different responses to stress. Specifically, while C3-KO cells were more sensitive to oxidative stress, C5-KO cells showed greater resistance. To explore this intriguing observation at the metabolic level, we evaluated anaerobic and aerobic glycolysis, along with mitochondrial function, in Lin^−^ BMMNC purified from C3-KO, C5-KO, and C5aR1-KO mice. We found that cells from complosome-deficient animals under steady-state conditions exhibited elevated lactate production and enhanced lactate dehydrogenase (LDH) release, indicating their reliance on anaerobic glycolysis. Interestingly, the uptake of a glucose fluorescent analog (2-NBDG) increased in C3-KO cells but decreased in C5-KO and C5aR1-KO cells compared to wild-type (WT) mice. Meanwhile, total ATP production in C3-KO cells, unlike that of C5 and C5aR1 mice, was reduced under steady-state conditions and did not change significantly after exposure to the mitochondrial-damaging agent hydrogen peroxide (H_2_O_2_). This suggests a greater dependence on anaerobic glycolysis in C3-KO cells than in C5-KO and C5aR1-KO cells. Finally, we assessed the integrity of mitochondrial membranes in the studied cells using MitoTracker green and deep red assays. Compared to WT cells, we observed that mitochondria from complosome mutant Lin-BMMNC accumulated fewer MitoTracker probes, indicating the presence of mitochondrial defects in these cells.

**Graphical Abstract:**

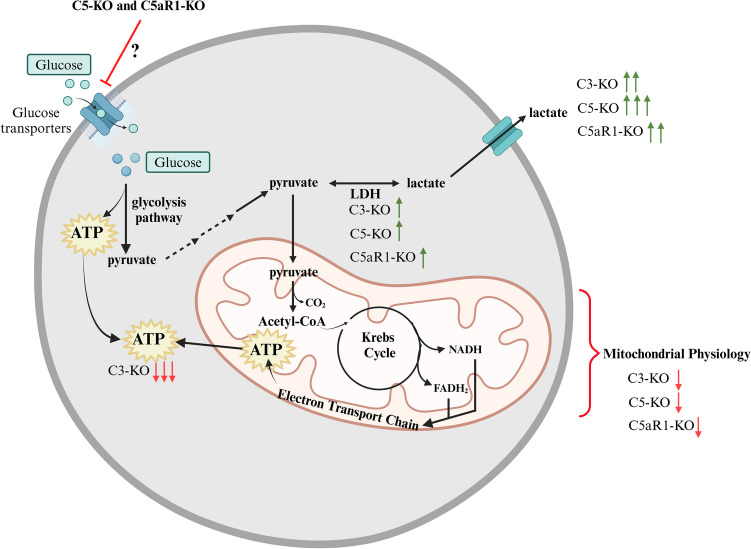

## Introduction

Traditionally recognized for its role in immune surveillance and host defense, the complement system has recently been found to play a crucial role inside cells. A groundbreaking study by Dr. Kemper and colleagues identified an intracellular network of complement activation, referred to as the "complosome," which regulates the biology of various cell types, including lymphocytes, innate immune cells, fibroblasts, endothelial cells, and epithelial cells [1–3]. This intracellular complement system is believed to control fundamental processes such as cell proliferation and survival, introducing a new paradigm for understanding complement biology beyond the classical, liver-derived complement proteins that circulate and activate in peripheral blood [1–3].

Recently, our group demonstrated that the complosome is expressed in hematopoietic stem/progenitor cells (HSPCs) and bone marrow (BM) stromal cells, playing a key role in regulating their trafficking, as evidenced by hematopoietic mobilization and homing/engraftment after transplantation [4]. The observation that complosome-deficient mice have fewer HSPCs and mesenchymal stem cells (MSCs) further confirms its impact on stem cell specification and proliferation [5]. Interestingly, we also found that C3-KO mice are more sensitive to oxidative stress compared to C5-KO mice [6]. Given that both mobilization and myeloablative conditions for transplantation through radio-chemotherapy induce a state of sterile inflammation in the BM microenvironment [7], this explains our findings regarding the poor mobilization in C5-KO and C5aR1-KO mice [8] and the delayed engraftment of their BM cells in wild-type recipients compared to those from C3-KO mice [9, 10].

This data can be explained by the fact that C3-KO deficient murine cells still express the C5 complosome protein, and its cleavage fragment C5a activates the intracellular C5aR1 on mitochondria [1–3]. Furthermore, the activation of the intracellular C5a-C5aR1 axis is crucial for stimulating the intracellular pattern recognition receptor (PRR)—Nlrp3 inflammasome, which positively regulates the trafficking of HSPCs, thereby promoting their mobilization, homing, and engraftment [11, 12]. In contrast, C5-KO mice or C5aR1-KO cells that do not respond to intracellular C5a express only C3, which supports the retention of HSPCs in the BM microenvironment [9], thereby not directly promoting their egress from the BM into PB in response to stress and pro-mobilizing agents.

To clarify these intriguing observations, this report focuses on the metabolism of C3-KO, C5-KO, and C5aR1-KO mice under steady-state conditions and in response to oxidative stress induced by hydrogen peroxide (H_2_O_2_). This research is supported by previous reports indicating that the intracellular complosome regulates the metabolism of lymphocytes and innate immune cells [1–3]. Here, we demonstrate for the first time that the complosome, like its role in lymphocytes, regulates the function of mitochondria in bone marrow-residing stem/progenitor cells, which provides a better explanation for the differences in stress responsiveness and metabolic consequences among C3-KO, C5-KO, and C5aR1-KO cells.

## Material and Methods

### Animals

Pathogen-free, 6–8-week-old female C57BL/6 J wild-type (WT), C3-KO (B6.129S4-*C3*^*tm1Crr*^/J, strain#029661), C5-KO (B10.D2-*Hc*^*0*^* H2*^*d*^* H2*-*T18*^*c*^/oSnJ, strain#000461), and C5aR1-KO (C.129S4(B6)-C5ar1tm1Cge/J, strain#033903) were acquired from the Jackson Laboratory (Bar Harbor, ME, USA) or the Central Laboratory for Experimental Animals at the Medical University of Warsaw. Prior to obtaining bone marrow, the mice were kept in an animal facility with a 12-h light/12-h dark cycle (lights on from 7:00 AM to 7:00 PM), and they had unlimited access to water and standard rodent food. All studies were conducted in accordance with the European Act on the protection of animals used for scientific or educational purposes and the guidelines set by the Animal Care and Use Committee of the Warsaw Medical University (Warsaw, Poland).

### Lineage-negative (Lin^−^) Cells Depletion

Lin^−^ cells were purified from WT, C3-KO, C5-KO, and C5aR1-KO mice using a Direct Lineage Cell Depletion Kit (Miltenyi Biotec, Bergisch Gladbach, Germany). Briefly, the cells were incubated for 10 min at 4 °C with MicroBeads conjugated to monoclonal antibodies against CD5, CD11b, CD45R (B220), Anti-Gr-1 (Ly-6G/C), 7–4, and Ter-119. Following incubation, the cells were washed with 3 ml of phosphate-buffered saline (PBS) and centrifuged at 1800 rpm for 10 min at 4 °C. The resulting cell pellet was resuspended in 1 ml of PBS, and the cell suspension was processed through an autoMACS™ Separator equipped with an autoMACS column (Miltenyi Biotec, Bergisch Gladbach, Germany) to isolate and collect the purified Lin⁻ cell population.

### Lactate Production Measurements

Lactate production in Lin^−^ cells isolated from WT, C3-KO, C5-KO, and C5aR1-KO BM was measured using the Lactate-Glo® Assay (Promega, Madison, WI, USA, Catalog No. J5021) following the manufacturer’s instructions. Control cells (untreated) and cells treated with H₂O₂ (10 µM) for 15 min were incubated at 37 °C in a humidified environment with 5% CO₂. After incubation, the cells were harvested, and 40,000 cells were seeded per well in a 96-well plate. Lactate-Glo® Reagent (50 µl/well) was added, and the plate was gently mixed for 45 s. The cells were then incubated at room temperature for 1 h. Luminescence was measured using a SpectraMax iD3 Multi-Mode Microplate Reader (Molecular Devices, CA, USA).

### Lactate Dehydrogenase (LDH) Release Measurements

LDH release in Lin^−^ cells from WT, C3-KO, C5-KO, and C5aR1-KO BM was assessed using the LDH-Glo™ Cytotoxicity Assay (Promega, Madison, WI, USA; Catalog No. J2380) according to the manufacturer’s instructions. Control (untreated) cells and cells treated with H₂O₂ (10 µM) for 15 min were incubated at 37 °C in a humidified atmosphere with 5% CO₂. After incubation, the cells were harvested, and 40,000 cells were resuspended in a suitable volume of phenol red-free RPMI medium. Cells were plated into a 96-well plate (50 µl per well). The LDH-Glo™ Reagent was added at a 1:1 ratio (50 µl/well), followed by gentle shaking for 1 min. The plate was then incubated at room temperature for 30 min. Luminescence intensity was measured using a SpectraMax iD3 Multi-Mode Microplate Reader (Molecular Devices, CA, USA) at 30, 45, and 60 min after incubation.

### Glucose Uptake Measurements

Glucose uptake was assessed using the fluorescent glucose analog 2-NBDG (2-[N-(7-Nitrobenz-2-oxa-1,3-diazol-4-yl)amino]-2-deoxyglucose, Thermo Fisher Scientific, Waltham, MA, USA). Lin^−^ cells isolated from WT, C3-KO, C5-KO, and C5aR1-KO bone marrow were pre-treated with either H₂O₂ (10 µM, 15 min), ATP (10 µM, 16 h), C5a (10 nM, 16 h), C3a (10 nM, 16 h). Next, cells were harvested and incubated with 2-NBDG (75 µM) in PBS (to minimize competition between glucose in the medium and 2-NBDG) for 30 min at 37 °C in a humidified atmosphere with 5% CO₂. After incubation, the cells were centrifuged at 1800 rpm for 10 min and washed three times with 1 ml of PBS. Following each wash, the cells were centrifuged under the same conditions. After the final wash, the cells were resuspended in an appropriate volume of PBS and seeded into a 96-well plate (40,000 cells in 100 µl PBS per well). Fluorescence was measured at λ_ex_ = 465 nm and λ_em_ = 540 nm using a SpectraMax iD3 Multi-Mode Microplate Reader (Molecular Devices, CA, USA).

### Total ATP Production

ATP production in Lin^−^ cells from WT, C3-KO, C5-KO, and C5aR1-KO BM was measured using the CellTiter-Glo® 2.0 Cell Viability Assay (Promega, Madison, WI, USA; Catalog No. G9241), following the manufacturer’s protocol. Briefly, Lin^−^ cells (40,000 cells per 100 µl of phenol red-free media) were pre-treated with either H₂O₂ (10 µM, 15 min), C5a (10 nM, 16 h), C3a (10 nM, 16 h), or 2-deoxy-D-glucose (2-DG, 2 mM, ThermoFisher Scientific, Waltham, MA, USA, 3.5 h) at 37 °C in a humidified atmosphere containing 5% CO₂. After incubation, the cells were centrifuged at 1800 rpm for 10 min, resuspended in an appropriate volume of phenol red-free RPMI medium, and plated into a 96-well plate (100 µl cells/well). Next, 100 µl of CellTiter-Glo reagent was added to each well and mixed gently for 3 min to ensure cell membrane lysis and ATP release. The cells were then incubated at room temperature for 10 min. Luminescence intensity, which indicates ATP levels, was measured using a SpectraMax iD3 Multi-Mode Microplate Reader (Molecular Devices, CA, USA) at 10, 20, and 30 min post-incubation.

### Mitochondrial Physiology Measurements

To assess mitochondrial physiology in wild-type (WT) and knockout mice, MitoTracker Deep Red and MitoTracker Green (ThermoFisher Scientific, Waltham, MA, USA) were utilized according to standard protocols. Briefly, Lin^−^ cells (40,000 cells per 100 µl of phenol red-free media (RPMI 1640 medium, no phenol red, ThermoFisher Scientific, Waltham, MA, USA)) were pre-treated with either H_2_O_2_ (10 µM, Merck, Darmstadt, Germany) for 15 min or with ATP (10 µM, Merck, Darmstadt, Germany), C3a (10 nM, Bio-Techne, MN, USA), and C5a (10 nM, Bio-Techne, MN, USA) for 16 h. After pre-treatment, the cells were centrifuged at 1800 rpm for 10 min. The resulting pellets were resuspended in 1 ml of phenol red-free RPMI medium containing either MitoTracker Deep Red or MitoTracker Green (final concentration: 50 pM). The cells were incubated at 37 °C in a humidified environment with 5% CO₂ for 15 min. Next, the cells were centrifuged at 1800 rpm for 10 min, followed by three sequential washes with 1 ml of phenol red-free RPMI medium. After each wash, the cells were centrifuged under the same conditions. Following the final wash, the cell pellets were resuspended in the appropriate volume of PBS. The resuspended cells (40,000 cells in 100 µl of media per well) were transferred to a 96-well plate. Fluorescence was measured using a SpectraMax iD3 Multi-Mode Microplate Reader (Molecular Devices, CA, USA). For MitoTracker Green, the excitation/emission wavelengths were λ_ex_ = 490 nm and λ_em_ = 516 nm, while for MitoTracker Deep Red, the excitation/emission wavelengths were λ_ex_ = 664 nm and λ_em_ = 665 nm.

### 2-NBDG Uptake Visualization and MitoTrackers Staining

Lin^−^ cells derived from murine bone marrow were incubated with or without H_2_O_2_ for 15 min as previously described. Cells were then treated with MitoTracker Deep Red (which targets the mitochondrial membrane), MitoTracker Green (which targets the mitochondrial cytosol), or 2-NBDG as mentioned earlier. Approximately 10,000 cells from each sample were placed on poly-L-lysine-coated slides in a Petri dish and transferred to a 37 °C, 5% CO_2_ cell culture incubator for 15 min. The cells were then fixed with a 4% paraformaldehyde (PFA) solution for 2 h, washed in PBS for 15 min, and stained with DAPI (Invitrogen, Life Technologies, MA, U.S.) for nuclear labeling for 15 min at room temperature. The slides were washed, and coverslips were mounted using mounting media (Dako, Glostrup, Denmark). Cells were imaged using a FluoView FV1000 laser-scanning confocal microscope (Olympus America Inc., Center Valley, PA, USA) to visualize the uptake of 2-NBDG, as well as MitoTracker Deep Red and MitoTracker Green.

### Statistical Analysis

All results are reported as the mean ± SD from at least three independent experiments. Statistical analyses were performed using GraphPad Prism 9.0 (GraphPad Software Inc., La Jolla, CA, USA). Data were analyzed using multiple unpaired t-tests, with significance set at *p* ≤ 0.05. Statistical significance is indicated as follows: * *p* < 0.05, ** *p* < 0.01, *** *p* < 0.001, **** *p* < 0.0001.

## Results

### Bone Marrow Stem Cells Isolated from C3-KO, C5-KO, and C5aR1-KO Mice Exhibited Higher Glycolytic Activity than those from WT Mice

Our previous findings identified impaired oxidative phosphorylation in Lin^−^ BMMNCs derived from complosome-deficient mice [6]. To better understand this phenomenon, we measured lactate production and evaluated the release of lactate dehydrogenase (LDH), the enzyme that converts pyruvate to lactate. These measurements were conducted under steady-state conditions and after the induction of mild oxidative stress following exposure to H_2_O_2_ [13, 14]. This approach allowed us to more thoroughly investigate the glycolytic pathway in these cells.

**Fig. 1:**
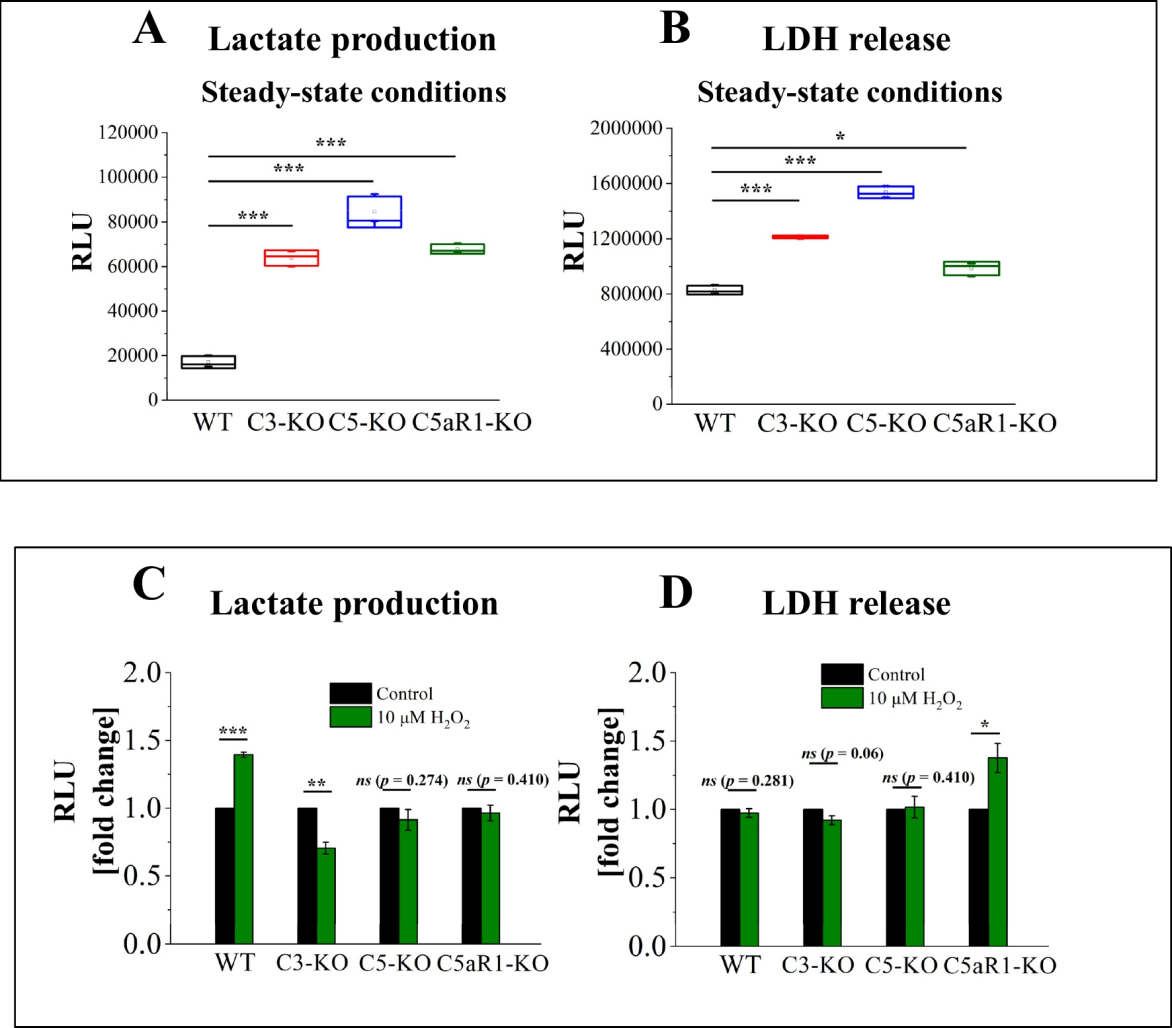
Glycolysis Efficiency Assessment– Lactate Production and LDH Release Measurements. Lactate production and LDH release are shown as relative luminescence units (RLU) to assess glycolytic activity in BM Lin⁻ stem cells isolated from WT, C3-KO, C5-KO, and C5aR1-KO mice exposed to 10 µM H₂O₂. Panels **A-B** compare lactate production (**A**) and LDH release (**B**) under steady-state conditions in cells from WT, C3-KO, C5-KO, and C5aR1-KO mice. Panels **C-D** illustrate lactate production (**C**) and LDH release (**D**) in cells treated with 10 µM H₂O₂, with results expressed as a percentage of the control (non-treated cells). Values are reported as the mean ± standard deviation (SD) from at least three independent experiments (*n* = 3). Statistical analysis was conducted using unpaired t-tests. Statistical significance is denoted by *p ≤ 0.05, **p ≤ 0.01, ***p ≤ 0.001

Figures 1A and B show that Lin^−^ BM stem cells isolated from C3-KO, C5-KO, and C5aR1-KO mice under steady-state conditions exhibited increased basal lactate production and LDH release, indicating elevated glycolysis in these mutant mice. Following the introduction of mild oxidative stress, distinct responses emerged among the groups. In C3-KO cells, lactate production significantly decreased after H₂O₂ treatment (Fig. 1C), suggesting a dependence on glycolysis for ATP production. In contrast, C5-KO and C5aR1-KO cells displayed no significant changes in lactate production (Fig. 1C), which reinforces previous findings that C5-KO mice have a greater capacity to adapt to oxidative stress [6].

Furthermore, in WT Lin^−^BMMNC, H₂O₂ treatment resulted in increased lactate production (Fig. 1C), indicating that during the early phase of mild oxidative stress, the shift in ATP production from oxidative phosphorylation to glycolysis serves as an adaptive response to mitochondrial damage. In contrast, no significant changes in LDH release were observed in WT, C3-KO, or C5-KO cells after H₂O₂ treatment (Fig. 1D). This suggests that the LDH release assay, commonly used as a cytotoxicity test, indicated these cells experienced mild oxidative stress without undergoing cell death. Conversely, H₂O₂ treatment of C5aR1-KO cells led to increased LDH release, a finding that warrants further investigation since lactate production remained unchanged under the same conditions.

### C3-KO Mice Exhibited an Opposite Effect on Glucose Uptake Compared to C5-KO and C5aR1-KO Mice

**Fig. 2:**
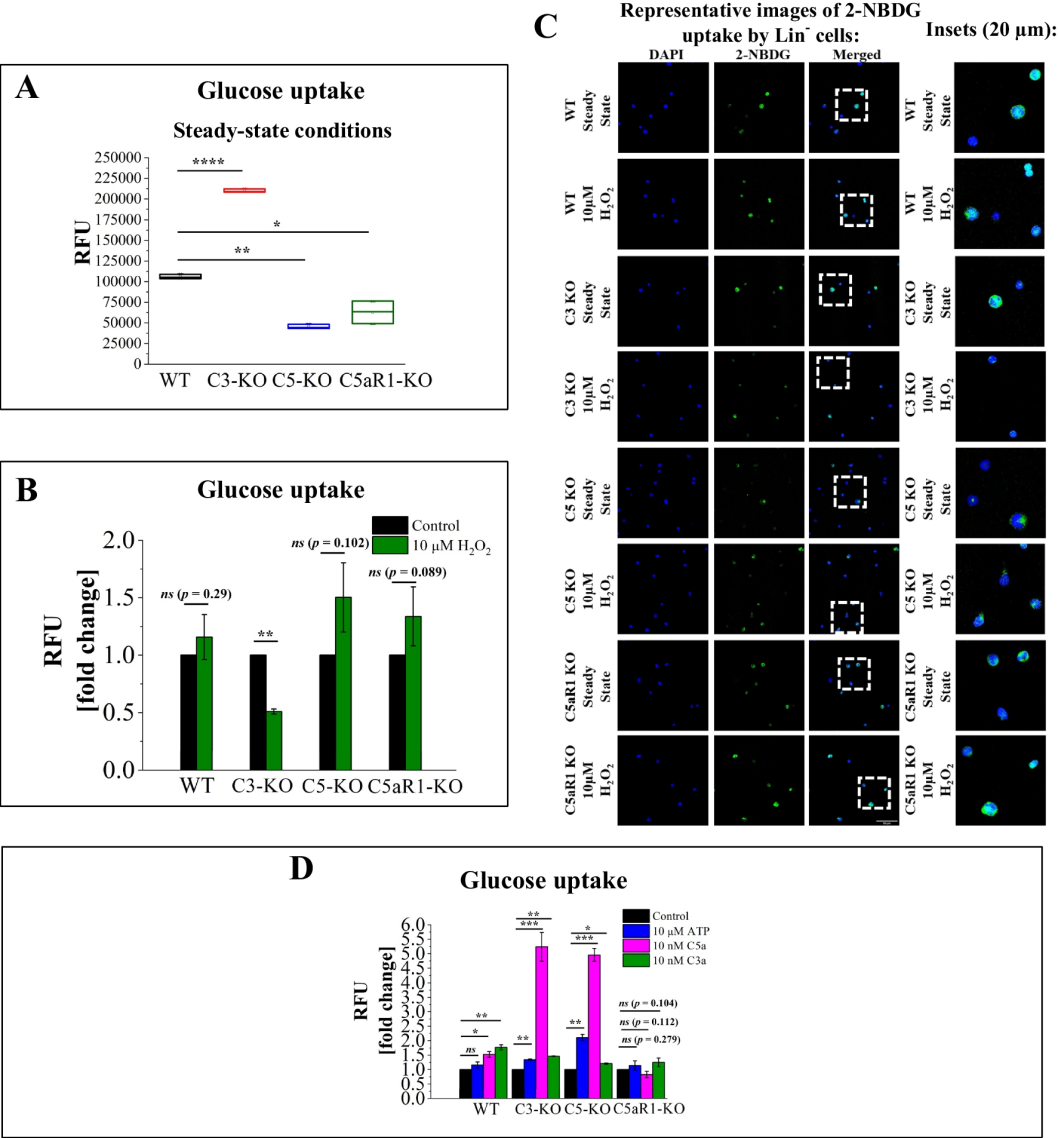
Glucose Uptake Measurements. Glucose uptake was evaluated in BM Lin⁻ stem cells isolated from WT, C3-KO, C5-KO, and C5aR1-KO mice under steady-state conditions or after exposure to 10 µM H₂O₂, 10 µM ATP, 10 nM C5a, or 10 nM C3a. Panel** A** displays relative fluorescence units (RFU) under steady-state conditions. Panel** B** shows RFU following treatment with 10 µM H₂O₂, expressed as a percentage of the non-treated control. Panel** C** includes representative confocal microscopy images of glucose uptake by Lin⁻ cells. Panel** D** presents RFU after stimulation with 10 µM ATP, 10 nM C5a, or 10 nM C3a, expressed as a percentage of the non-treated control. Values are presented as the mean ± standard deviation (SD) from at least three independent experiments (*n* = 3). Statistical analysis was conducted using unpaired t-tests. Statistical significance is indicated by *p ≤ 0.05, **p ≤ 0.01, ***p ≤ 0.001

We hypothesized that the observed increase in glycolysis would correlate with enhanced glucose uptake [15]. To verify this, we used the fluorescent glucose analog 2-NBDG, which enters cells through the same transport mechanisms as glucose [16, 17]. However, unlike glucose-6-phosphate, the phosphorylated form of 2-NBDG cannot be further metabolized in glycolysis, leading to its accumulation in the cytoplasm. This accumulation allows visualization through fluorescence/confocal microscopy or spectrofluorometry. Our findings show that, under steady-state conditions, only C3-KO Lin⁻ BM stem cells exhibited increased glucose uptake (Figs. 2A and C). Surprisingly, despite having higher basal glycolytic activity compared to WT cells, both C5-KO and C5aR1-KO cells showed reduced glucose uptake. This reduction may result from impaired glucose transporters (GLUTs) in these cells [18], a hypothesis that will be explored in future studies. Confocal microscopy clearly illustrates variability in the ability of lineage-negative cell subpopulations to accumulate 2-NBDG, highlighting differences in GLUT activity among these subpopulations (Fig. 2C). We will consider this finding in future studies focusing on more purified cell populations.

Next, we examined whether H₂O₂ treatment influences glucose uptake. In WT, C5-KO, and C5aR1-KO cells, we noted a slight but statistically insignificant increase in glucose uptake (Fig. 2B). This trend may suggest an adaptive response to mild oxidative stress, where cells shift energy production toward glycolysis due to mitochondrial damage. In contrast, C3-KO cells exhibited nearly a two-fold reduction in glucose uptake compared to basal conditions. We hypothesize that these cells have impaired mitochondrial function and lack adaptive mechanisms to cope with oxidative stress, making them more susceptible to damage [6].

We also investigated the effects of ATP, C5a, and C3a stimulation on glucose uptake (Fig. 2D). Treatment with ATP and C3a enhanced glucose uptake in WT, C3-KO, and C5-KO cells. However, stimulation with C5a in cells from C3-KO and C5-KO mice resulted in nearly a six-fold increase in glucose uptake. Since C5aR1-KO cells exhibited no increase in glucose uptake in response to C5a, this suggests the involvement of C5aR1 rather than C5aR2 in this phenomenon.

### Measurements of ATP Production in Lin^−^ BMMNC Isolated from Wild-type (WT) and Complosome-deficient Mice

**Fig. 3:**
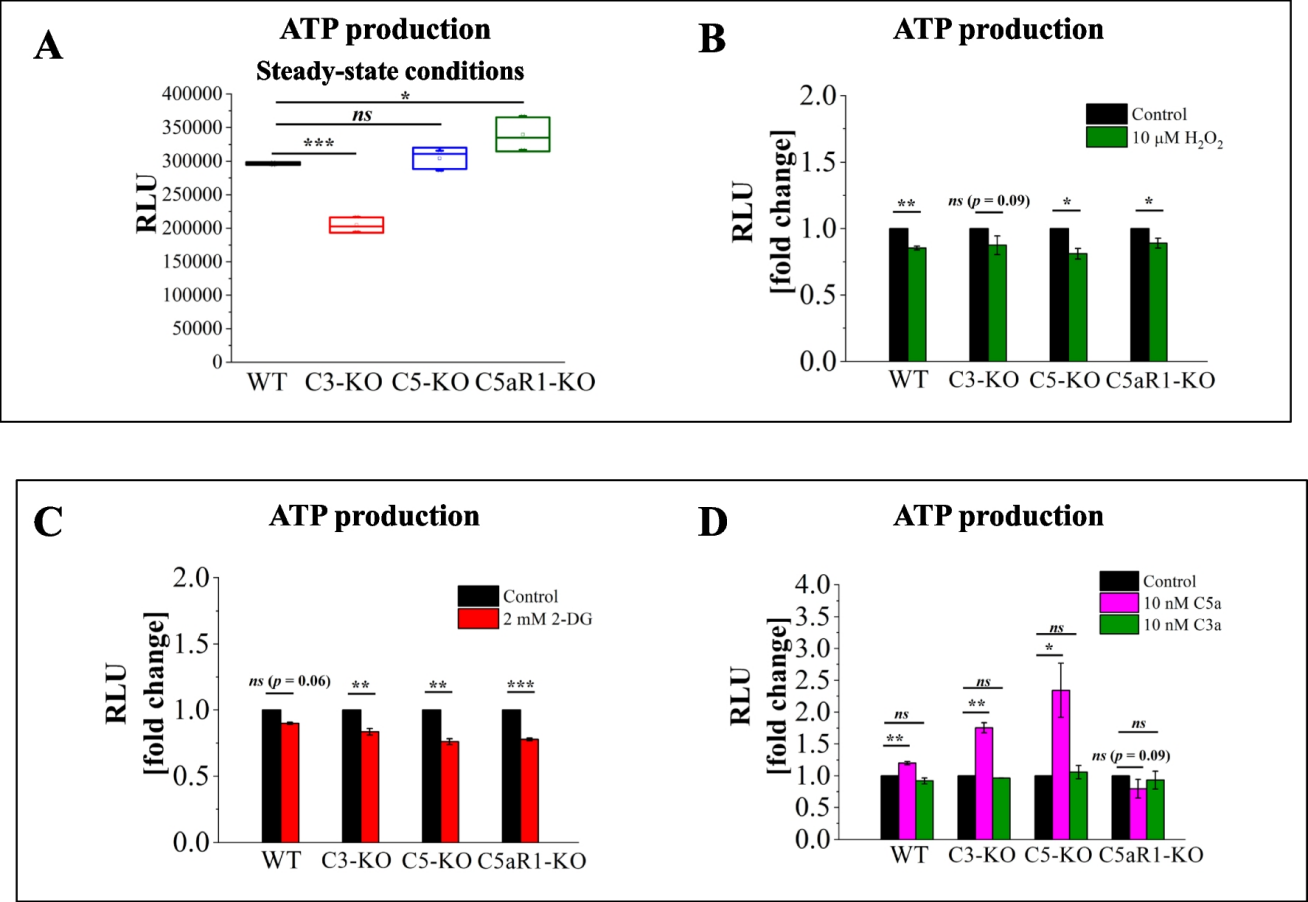
ATP Production in Lin⁻ BMMNC. ATP production was measured in BM Lin⁻ stem cells isolated from WT, C3-KO, C5-KO, and C5aR1-KO mice under steady-state conditions or after exposure to 10 µM H₂O₂, 2 mM 2-DG, 10 nM C5a, or 10 nM C3a. Panel **A**: Relative luminescence units (RLU) under steady-state conditions. Panel** B**: RLU following treatment with 10 µM H₂O₂, represented as a percentage of the non-treated control. Panel** C**: RLU measured after treatment with the glycolysis inhibitor 2-DG (2 mM), represented as a percentage of the control. Panel** D**: RLU after stimulation with 10 nM C5a or 10 nM C3a, represented as a percentage of the control. Values are reported as the mean ± standard deviation (SD) from at least three independent experiments (*n* = 3). Statistical analysis was conducted using unpaired t-tests. Statistical significance is indicated by *p ≤ 0.05, **p ≤ 0.01, ***p ≤ 0.001

Based on our previous data, we assumed that C3-KO cells might exhibit impaired oxidative phosphorylation. We hypothesized that if ATP production shifts toward glycolysis and alternative pathways, the overall ATP levels in these cells would be lower compared to WT cells. To test this hypothesis, we utilized the CellTiter-Glo 2.0 assay. Under steady-state conditions, C3-KO cells showed lower ATP levels, further supporting mitochondrial dysfunction. In contrast, C5-KO and C5aR1-KO cells maintained ATP levels similar to those of WT cells, with a slight increase observed in C5aR1-KO cells (Fig. 3A). Figure 3B illustrates ATP production following exposure to H₂O₂. In this experiment, where H₂O₂ exposure primarily damages mitochondria, ATP production remained stable in C3-KO cells, confirming that these cells primarily rely on anaerobic glycolysis rather than mitochondrial ATP production. In the subsequent experiment, we treated cells with the glycolysis inhibitor 2-DG and, as anticipated, observed a decrease in ATP production across all cell types (Fig. 3C). Lastly, we examined the effect of C3a and C5a stimulation on ATP production (Fig. 3D). C3a treatment did not impact ATP production in any of the treated cells. However, C5a treatment resulted in increased ATP production in WT, C3-KO, and C5-KO cells, with the most pronounced effect noted in C5-KO cells. Meanwhile, cells isolated from C5aR1-KO mice did not respond to C5a treatment, which aligns with our glucose uptake measurements and indicates a role for C5aR1 in this phenomenon.

### Investigation of Mitochondrial Physiology with MitoTrackers

**Fig. 4:**
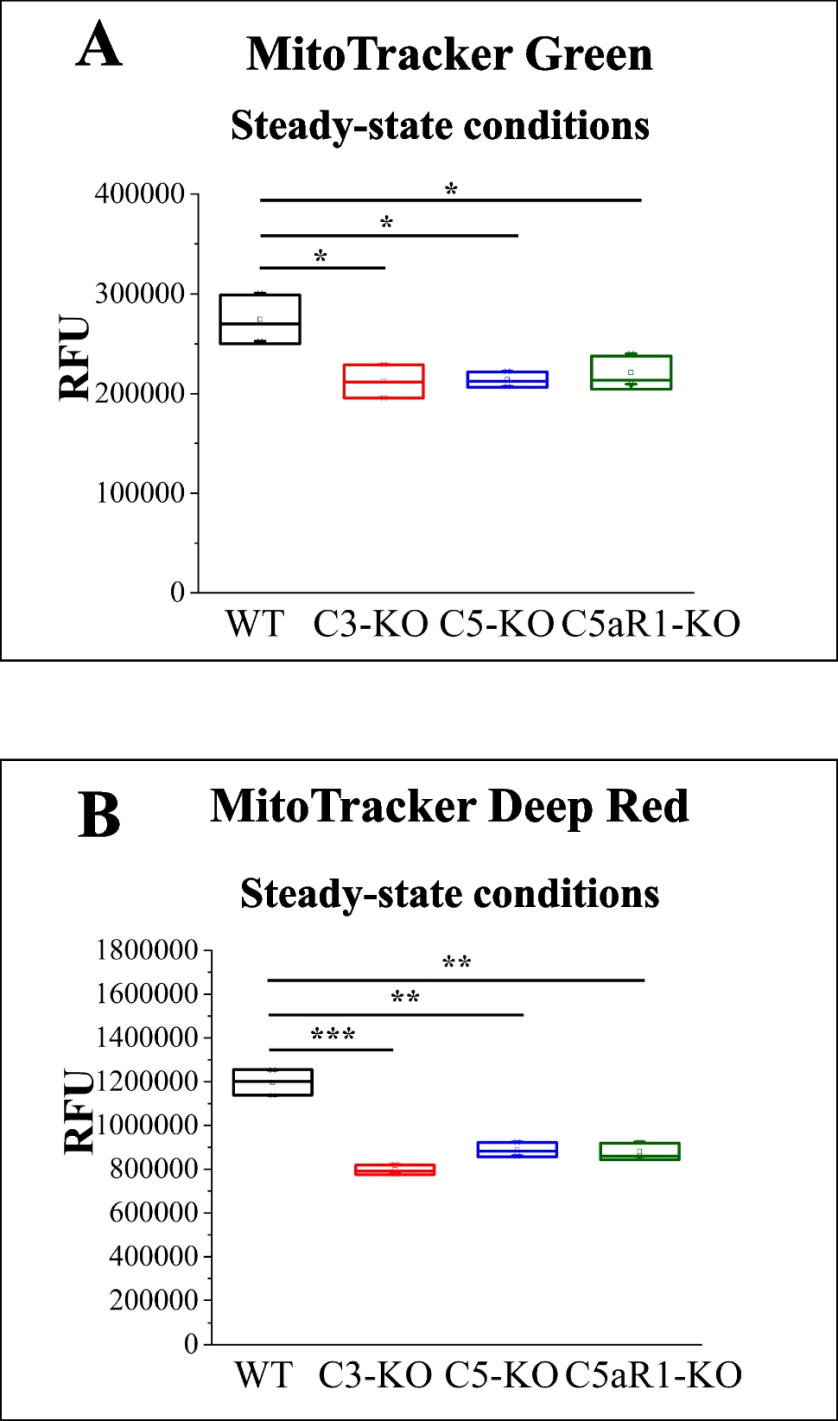
Assessment of Mitochondrial Physiology in Lin⁻ BMMNC. Mitochondrial physiology was assessed in BM Lin⁻ stem cells isolated from WT, C3-KO, C5-KO, and C5aR1-KO mice under steady-state conditions. Panel** A**: Relative fluorescence units (RFU) of MitoTracker Green, which indicates overall mitochondrial morphology. Panel** B**: RFU of MitoTracker Deep Red, reflecting mitochondrial membrane potential. Values are reported as the mean ± standard deviation (SD) from at least three independent experiments (*n* = 3). Statistical analysis was conducted using unpaired t-tests, with statistical significance denoted by *p ≤ 0.05, **p ≤ 0.01, ***p ≤ 0.001

To assess mitochondrial physiology, we used two mitochondrial dyes: MitoTracker Green and MitoTracker Deep Red. Both dyes selectively target mitochondria, but their accumulation mechanisms differ. MitoTracker Green localizes within the mitochondrial matrix, providing insight into overall mitochondrial morphology [19, 20]. In contrast, MitoTracker Deep Red accumulates in the mitochondrial membrane in a membrane potential-dependent manner, labeling only those mitochondria that maintain an intact membrane potential [19, 20]. We first evaluated mitochondrial physiology in each mutant under steady-state conditions (Figs. 4A and B). In both cases, the fluorescence intensity was lower in mutant mice compared to WT for both MitoTracker Green and Deep Red. These findings strongly suggest mitochondrial dysfunction in cells lacking complement components C3, C5, or the C5aR1 receptor.

**Fig. 5:**
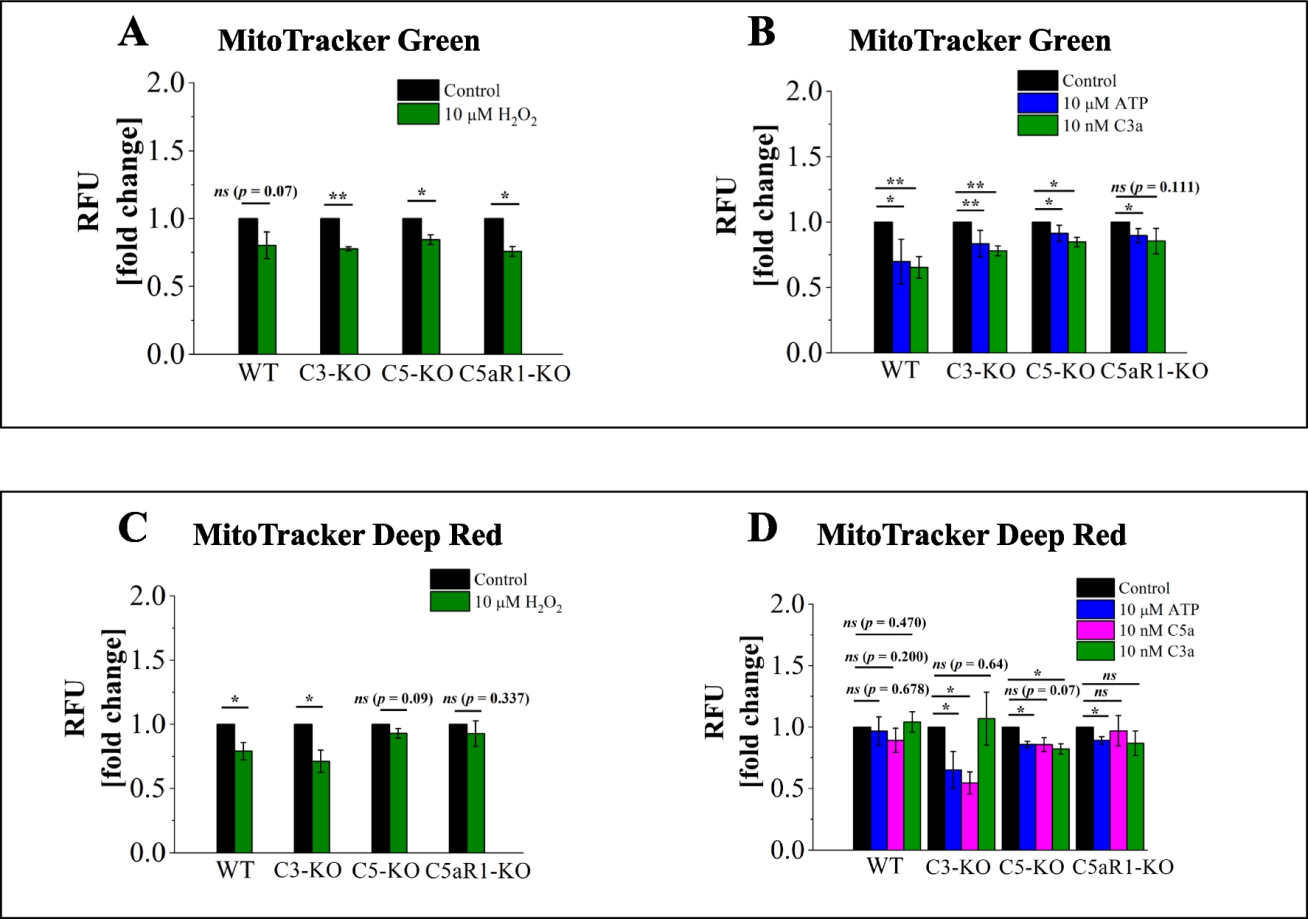
Mitochondrial Analysis in Lin⁻ BMMNC. Mitochondrial function was evaluated in BM Lin⁻ stem cells isolated from WT, C3-KO, and C5-KO mice after treatment with 10 µM H₂O₂ or stimulation with 10 µM ATP, 10 nM C5a, or 10 nM C3a. Panel** A**: Relative fluorescence units (RFU) of MitoTracker Green in cells treated with 10 µM H₂O₂, indicating overall mitochondrial morphology. Panel** B**: RFU of MitoTracker Green in cells after stimulation with 10 µM ATP or 10 nM C3a. Panel** C**: RFU of MitoTracker Deep Red in cells following 10 µM H₂O₂ treatment, reflecting mitochondrial membrane potential. Panel** D**: RFU of MitoTracker Deep Red in cells treated with 10 µM ATP, 10 nM C5a, or 10 nM C3a. All values are presented as the mean ± standard deviation (SD) from at least three independent experiments (*n* = 3). Statistical analysis was conducted using unpaired t-tests. Statistical significance is denoted by *p ≤ 0.05, **p ≤ 0.01, ***p ≤ 0.001

**Fig. 6:**
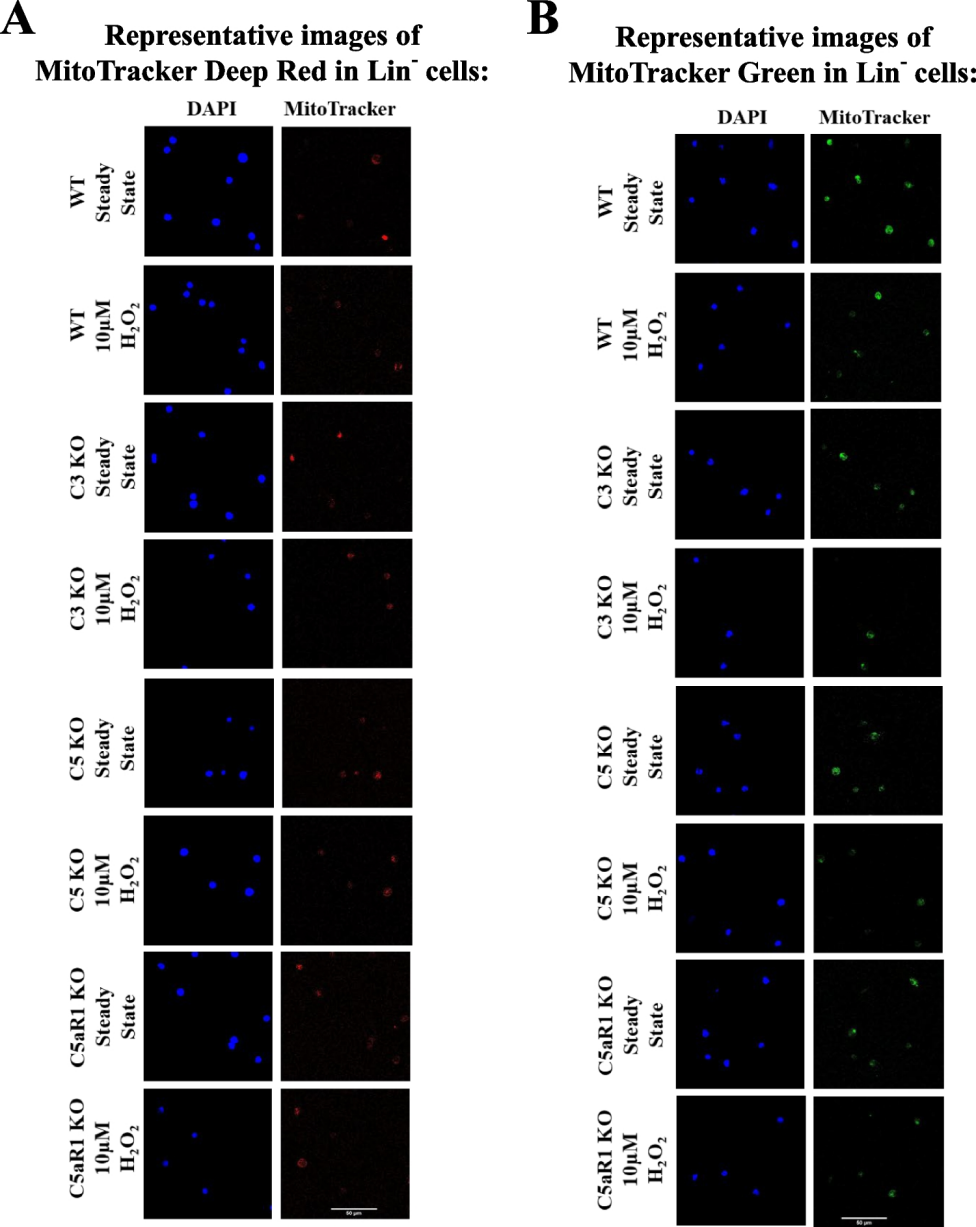
Mitochondrial Membrane Potential in Lin⁻ BMMNC. Representative confocal images of Lin^−^ cells derived from the bone marrow of WT, C3-KO, C5-KO, and C5aR1-KO mice under steady-state conditions and after treatment with 10 µM H₂O₂, as indicated. (**A**) Mitochondrial membrane potential was evaluated using Mitotracker Deep Red (red), which accumulates in mitochondria with intact membrane potential. (**B**) Mitochondrial morphology was examined using Mitotracker Green (green), which stains the mitochondrial matrix. Both Mitotracker dyes were introduced into live cells prior to fixation. Fixed cells were co-stained with DAPI (blue) for nuclear visualization. Scale bar: 50 µm

Next, we examined the effects of mild oxidative stress (H₂O₂ treatment) on mitochondria. MitoTracker Green fluorescence intensity decreased uniformly across all studied groups, indicating that H₂O₂ induces similar intracellular mitochondrial changes (Fig. 5A). However, MitoTracker Deep Red showed that stem cells isolated from C5-KO and C5aR1-KO mice maintained their mitochondrial membrane potential, as no significant decrease in fluorescence was observed (Fig. 5C). In contrast, WT and C3-KO cells exhibited a notable reduction in fluorescence, suggesting destabilization of the mitochondrial membrane (Fig. 5C). To further illustrate these effects, we performed staining and visualized the results using confocal microscopy (Fig. 6).

In the final step, we stimulated Lin⁻ stem cells overnight with ATP, C3a, and C5a, and then assessed mitochondrial physiology using both dyes (Figs. 5B and D). MitoTracker Green staining exhibited a change in mitochondrial physiology following stimulation, with the most pronounced effect observed in WT cells. This finding aligns with our previous study, which demonstrated that ATP, C5a, and C3a stimulation increases ROS production [6], potentially impairing mitochondrial function, as shown here. However, in WT cells, this impairment did not affect mitochondrial membrane potential, as the MitoTracker Deep Red fluorescence intensity remained unchanged after stimulation (Fig. 5D). Meanwhile, Lin⁻ BMMNC from C5-KO and C5aR1-KO mice showed a slight decrease in fluorescence intensity, suggesting that stimulation did not significantly destabilize the mitochondrial membrane potential in these cells (Fig. 5D). In contrast, the most notable disruption of membrane potential occurred in C3-KO cells, where ATP and C5a treatment led to nearly a twofold decrease in fluorescence intensity. Notably, at the same time, C3-KO cells did not demonstrate membrane depolarization in response to C3a stimulation (Fig. 5D).

## Discussion

This study's key finding is that murine Lin^−^BMMNCs isolated from C3-, C5-, or C5aR1-deficient mice, enriched in hematopoietic stem/progenitor cells, exhibit a functional mitochondrial defect. Consequently, our data align with the mitochondrial defects observed in complosome-deficient lymphocytes [1–3]. In our metabolic studies, we also noted a greater reliance on anaerobic glycolysis in C3-KO cells compared to C5-KO and C5aR1-KO cells. This divergence highlights functional differences in how individual complosome components regulate cellular metabolism and stress responses, underscoring the complexity of the intracellular complement network.

In our previous paper, we reported that C3-KO mice, unlike C5-KO animals, exhibit increased sensitivity to oxidative stress and enhanced activation of the intracellular Nox2-ROS-Nlrp3 inflammasome signaling pathway [6]. This explains the differences in mobilization and homing/engraftment efficacy noted between C3-KO and C5-KO murine strains [8–10]. However, since both strains displayed a reduced oxygen consumption rate under steady-state conditions in our Seahorse analysis [6], it suggests that these mutant animals may also face mitochondrial defects. This issue has been examined in greater detail in the current work.

Evidence is accumulating that innate immunity and cell metabolism are closely linked, with both extracellular and intracellular complements playing crucial roles in coordinating these processes [4, 21–23]. Based on research conducted on human lymphocytes, Kemper et al. proposed that a deficiency of intracellular complement proteins leads to mitochondrial dysfunction [1–3]. Mitochondria are suggested to be the preferred organelles for complement [24], and both mitochondria and lysosomes, which are essential components of cellular metabolic machinery, serve as hotspots of complosome activity [3].

Hematopoietic and lymphopoietic cells arise from the same multipotent stem cells, so it is not surprising that both cell types express similar genes, including those that encode the complosome and the pattern recognition receptor Nlrp3 inflammasome, as we reported [4, 11, 12, 25]

In our experiments, C3-KO mice express the intracellular component of the C5 complosome, while C5-KO cells express the C3 complosome protein. These components play a role in regulating cell biology, and several intracellular organelles, including mitochondria, have receptors for the C3 and C5 cleavage fragments, C3a and C5a– specifically C3aR and C5aR1. Furthermore, murine and human HSPCs express mRNA for C5aR2 [4].

C3a and C5a, as intracellular complosome mediators, likely play similar yet distinct roles in cell biology. Our previous data on the sensitivity of complosome-deficient mutants to stressful conditions, along with the differences noted in our current studies at the metabolic level, support this. We also observed that C5-KO mice have fewer HSPCs, MSCs, and very small embryonic-likes stem cells (VSELs) in the bone marrow [5], in contrast to C3-KO animals. Additionally, this work highlights the differences in sensitivity to stress between complosome-deficient cells, indicating that C3-KO cells rely more on anaerobic glycolysis under steady-state conditions than their C5-KO and C5aR1-KO counterparts. However, when mild oxidative stress occurs, the resistance observed in C5-KO mice can be attributed to the already heightened glycolysis in these mice. When oxidative phosphorylation is impaired, ATP production shifts to glycolysis, similar to the process in C3-KO cells under steady-state conditions. Furthermore, we found that C5a, unlike C3a, enhances glucose uptake and ATP production in C3-KO and C5-KO Lin^−^ BMMNC. Additionally, since C5a activates two receptors, C5aR1 and C5aR2, our data with C5aR1-KO cells indicate that these effects are mediated by C5aR1, as they were abolished in Lin^−^ BMMNC from these animals.

We noticed that C5-KO and C5aR1-KO cells have a decrease in glucose uptake, which could indicate a defect in glucose transporters. To support this, it has been reported that the complosome plays a role in glucose uptake in lymphocytes by regulating the expression of the Glut-1 glucose transporter and modulating the expression of several enzymes involved in metabolism [1–3, 24]. This mechanism is attributed to cleaved signaling fragments of CD46, known as CYT-1 and CYT-2, which, upon activation by C3a, translocate to the nucleus and directly regulate gene expression at the DNA level. [3, 26, 27]. While this mechanism functions in human cells, it may not apply to mice. It has been reported that rodents do not express CD46 in somatic tissues, and a functional murine homologue serving the various roles of CD46 has not yet been identified [3, 28]. Therefore, the potential molecular mechanism of C5a in the expression of glucose transporters and the regulation of several metabolic enzymes, as previously reported [4, 11, 12], requires further molecular studies.

We know that the Lin^−^ BMMNC population is enriched with HSPCs and other cells that comprise the hematopoietic BM microenvironment. Therefore, further studies on a more purified population of HSPCs and BM-purified MSCs will be necessary. To support the role of the hematopoietic microenvironment, we previously reported that both C3-KO [10] and C5-KO [8] mice exhibit microenvironmental defects in the homing and engraftment of normal BM cells that were transplanted.

Further investigation is also needed to understand how the complosome affects cell metabolism. There are three major possibilities. First, it can directly regulate the activity of these organelles by engaging receptors expressed on their surface. Second, the complosome may influence the expression of genes that regulate metabolic pathways at the nuclear level. Finally, the most significant effect could be modulating the ROS-Nlrp3 inflammasome axis. ROS are well-known for oxidizing cysteine and methionine residues in proteins, which modifies enzymes, membrane transporters, structural proteins, and histones [29, 30]. We observed that Nox2-induced ROS “redox signaling” modulates the expression of several enzymes involved in the metabolism of lipids, amino acids, and glucose [31].

In conclusion, our data is essential for enhancing the understanding of the biological effects of complosome. Our results suggest that complosome may have both similar and distinct roles in regulating biology in lymphocytes and hematopoietic cells. This is emphasized by the fact that, although these two types of cells share a common developmental origin, they are involved in different biological processes.

## Data Availability

Detailed data is available upon request.
